# Supplementary material for: Double extreme-cum-median ranked set sampling
Source: PLoS One. 2024 Dec 19;19(12):e0312140. doi: 10.1371/journal.pone.0312140 (PMC11658490; doi:10.1371/journal.pone.0312140)
Supplement: S1 Appendix — (DOCX) [file pone.0312140.s001.docx]

**Appendix I**

*For even sample size (Equation 12)*

$$\mu_{y,DEMRSS}^{(E)}=\sum_{j=1}^{r} \left[ \sum_{i=1}^{\frac{m}{2}} Y_{1\left( 1:4 \right)j}^{i\left( 1:4 \right)}+\sum_{i=\frac{m}{2}+1}^{m} Y_{2m\left( 4:4 \right)j}^{i\left( 4:4 \right)}+\sum_{i=m+1}^{\frac{3m}{2}} Y_{m\left( 2:4 \right)j}^{i\left( 2:4 \right)}+\sum_{i=\frac{3m}{2}+1}^{2m} Y_{\left( m+1 \right)\left( 3:\$ \right)j}^{i\left( 3:4 \right)} \right]$$

Or

$$\bar{y}_{,DEMRSS}^{(E)}=\sum_{j=1}^{r} \left[ \frac{\left( \sum_{i=1}^{\frac{m}{2}} Y_{1\left( 1:4 \right)j}^{i\left( 1:4 \right)}+\sum_{i=\frac{m}{2}+1}^{m} Y_{2m\left( 4:4 \right)j}^{i\left( 4:4 \right)} \right)+\left( \sum_{i=m+1}^{\frac{3m}{2}} Y_{m\left( 2:4 \right)j}^{i\left( 2:4 \right)}+\sum_{i=\frac{3m}{2}+1}^{2m} Y_{\left( m+1 \right)\left( 3:4 \right)j}^{i\left( 3:4 \right)} \right)}{2rm} \right]$$

Apply variance

$$var\left( \bar{y}_{,DEMRSS}^{\left( E \right)} \right)=\frac{1}{4\left( rm \right)^{2}}\left[ \sum_{j=1}^{r} \left\{ \left( \sum_{i=1}^{\frac{m}{2}} {\sigma^{2}}_{1\left( 1 \right)j}^{i\left( 1 \right)}+\sum_{i=\frac{m}{2}+1}^{m} {\sigma^{2}}_{2m\left( 2m \right)j}^{i\left( 2m \right)} \right)+\left( \sum_{i=m+1}^{\frac{3m}{2}} {\sigma^{2}}_{m\left( m \right)j}^{i\left( m \right)}+\sum_{i=\frac{3m}{2}+1}^{2m} {\sigma^{2}}_{\left( m+1 \right)\left( m+1 \right)j}^{i\left( m+1 \right)} \right) \right\} \right]$$

$$var\left( \bar{y}_{,DEMRSS}^{\left( E \right)} \right)=\frac{1}{4\left( rm \right)^{2}}\left[ r\left( {\frac{m}{2}\sigma^{2}}_{1\left( 1 \right)}^{\left( 1 \right)}+\frac{m}{2}{\sigma^{2}}_{2m\left( 2m \right)}^{\left( 2m \right)} \right)+\left( \frac{m}{2}{\sigma^{2}}_{m\left( m \right)}^{\left( m \right)}+\frac{m}{2}{\sigma^{2}}_{\left( m+1 \right)\left( m+1 \right)}^{\left( m+1 \right)} \right) \right]$$

$$=\frac{1}{4\left( rm \right)^{2}}\left[ \frac{rm}{2}\left\{ \left( {\sigma^{2}-\varphi^{2}}_{1\left( 1 \right)}^{\left( 1 \right)}+{\sigma^{2}-\varphi^{2}}_{2m\left( 2m \right)}^{\left( 2m \right)} \right)+\left( {\sigma^{2}-\varphi^{2}}_{m\left( m \right)}^{\left( m \right)}+{\sigma^{2}-\varphi^{2}}_{\left( m+1 \right)\left( m+1 \right)}^{\left( m+1 \right)} \right) \right\} \right]$$

$$=\frac{1}{4\left( rm \right)^{2}}\left[ \frac{rm}{2}\left( 4{\sigma^{2}-\varphi^{2}}_{1\left( 1 \right)}^{\left( 1 \right)}-{\varphi^{2}}_{2m\left( 2m \right)}^{\left( 2m \right)}{-\varphi^{2}}_{m\left( m \right)}^{\left( m \right)}{-\varphi^{2}}_{\left( m+1 \right)\left( m+1 \right)}^{\left( m+1 \right)} \right) \right]$$

$$\frac{\sigma^{2}}{2rm}-\frac{1}{4{rm}^{2}}\left[ \frac{m}{2}\left\{ \left( {\varphi^{2}}_{1\left( 1 \right)}^{\left( 1 \right)}+{\varphi^{2}}_{m\left( m \right)}^{\left( m \right)} \right)+\frac{m}{2}\left( {\varphi^{2}}_{m\left( m \right)}^{\left( m \right)}+{\varphi^{2}}_{\left( m+1 \right)\left( m+1 \right)}^{\left( m+1 \right)} \right) \right\} \right]$$

*For odd sample size (Equation 13)*

From the first cycle, we got $Y_{1\left( 1:6 \right)1}^{\left( 1 \right)\left( 1:6 \right)}$, $Y_{6\left( 6:6 \right)1}^{\left( 2 \right)\left( 6:6 \right)}$, $Y_{3\left( 3:6 \right)1}^{\left( 3 \right)\left( 3:6 \right)}$, $Y_{4\left( 4:6 \right)1}^{\left( 4 \right)\left( 4:6 \right)}$, $Y_{3\left( 3:6 \right)1}^{\left( 5 \right)\left( 3:6 \right)}$ and $Y_{4\left( 4:6 \right)1}^{\left( 6 \right)\left( 4:6 \right)}$.

On the same lines, from the second cycle, we will select $Y_{1\left( 1:6 \right)2}^{\left( 1 \right)\left( 1:6 \right)}$, $Y_{6\left( 6:6 \right)2}^{\left( 2 \right)\left( 6:6 \right)}$, $Y_{3\left( 3:6 \right)2}^{\left( 3 \right)\left( 3:6 \right)}$, $Y_{4\left( 4:6 \right)2}^{\left( 4 \right)\left( 4:6 \right)}$, $Y_{3\left( 3:6 \right)2}^{\left( 5 \right)\left( 3:6 \right)}$ and $Y_{4\left( 4:6 \right)2}^{\left( 6 \right)\left( 4:6 \right)}Y_{4\left( 4:6 \right)2}^{\left( 6 \right)\left( 4:6 \right)}$.

$$\mu_{y,DEMRSS}^{\left( O \right)}=\frac{1}{2rm}\sum_{j=1}^{r} \left[ \begin{aligned} &\left( \sum_{i=1}^{\frac{\left( m-1 \right)}{2}} Y_{1\left( 1 \right)j}^{i\left( 1 \right)}+\sum_{i=\left\{ \frac{\left( m-1 \right)}{2} \right\}+1}^{m-1} Y_{2m\left( 2m \right)j}^{i\left( 2m \right)}+Y_{m\left( m \right)j}^{m\left( m \right)} \right) \\ &+\left( Y_{\left( m+1 \right)\left( m+1 \right)j}^{\left( m+1 \right)\left( m+1 \right)}+\sum_{i=m+2}^{\frac{\left( 3m+1 \right)}{2}} Y_{m\left( m \right)j}^{i\left( m \right)}+\sum_{i=\frac{3\left( m+1 \right)}{2}}^{2m} Y_{\left( m+1 \right)\left( m+1 \right)j}^{i\left( m+1 \right)} \right) \end{aligned} \right]$$

Apply variance

$$var\left( \bar{y}_{,DEMRSS}^{\left( O \right)} \right)=\frac{1}{4\left( rm \right)^{2}}\left[ r\left( {\frac{m-1}{2}\sigma^{2}}_{1\left( 1 \right)j}^{\left( 1 \right)}+\frac{m-1}{2}{\sigma^{2}}_{2m\left( 2m \right)j}^{\left( 2m \right)}+{\sigma^{2}}_{m\left( m \right)j}^{\left( m \right)} \right)+\left( {\sigma^{2}}_{\left( m+1 \right)\left( m+1 \right)j}^{\left( m+1 \right)}+\frac{m-1}{2}{\sigma^{2}}_{m\left( m \right)j}^{\left( m \right)}+\frac{m-1}{2}{\sigma^{2}}_{\left( m+1 \right)\left( m+1 \right)j}^{\left( m+1 \right)} \right) \right]$$

$$=\frac{1}{4\left( rm \right)^{2}}\left[ r\left\{ \frac{m-1}{2}\left( {\sigma^{2}-\varphi^{2}}_{1\left( 1 \right)}^{\left( 1 \right)} \right)+\frac{m-1}{2}\left( {\sigma^{2}-\varphi^{2}}_{2m\left( 2m \right)}^{\left( 2m \right)} \right)+\left( {\sigma^{2}-\varphi^{2}}_{m\left( m \right)}^{\left( m \right)} \right)+\left( {\sigma^{2}-\varphi^{2}}_{\left( m+1 \right)\left( m+1 \right)}^{\left( m+1 \right)} \right)+\frac{m-1}{2}\left( {\sigma^{2}-\varphi^{2}}_{m\left( m \right)}^{\left( m \right)} \right)+\frac{m-1}{2}\left( {\sigma^{2}-\varphi^{2}}_{\left( m+1 \right)\left( m+1 \right)}^{\left( m+1 \right)} \right) \right\} \right]$$

$$=\frac{1}{4\left( rm \right)^{2}}\left[ r\left\{ \frac{2\left( m-1 \right)}{2}\sigma^{2}-\frac{m-1}{2}\left( {\varphi^{2}}_{1\left( 1 \right)}^{\left( 1 \right)}{+\varphi^{2}}_{2m\left( 2m \right)}^{\left( 2m \right)} \right)+2\sigma^{2}-\left( {\varphi^{2}}_{m\left( m \right)}^{\left( m \right)}+{\varphi^{2}}_{\left( m+1 \right)\left( m+1 \right)}^{\left( m+1 \right)} \right)-\frac{m+1}{2}\left( {\varphi^{2}}_{m\left( m \right)}^{\left( m \right)}+{\varphi^{2}}_{\left( m+1 \right)\left( m+1 \right)}^{\left( m+1 \right)} \right) \right\} \right]$$

$$=\frac{1}{4\left( rm \right)^{2}}\left[ r\left\{ {2m\sigma}^{2}-\left( \frac{m-1}{2}\left( {\varphi^{2}}_{1\left( 1 \right)}^{\left( 1 \right)}{+\varphi^{2}}_{2m\left( 2m \right)}^{\left( 2m \right)} \right)+\frac{m+1}{2}\left( {\varphi^{2}}_{m\left( m \right)}^{\left( m \right)}+{\varphi^{2}}_{\left( m+1 \right)\left( m+1 \right)}^{\left( m+1 \right)} \right) \right) \right\} \right]$$

$$=\frac{1}{4\left( rm \right)^{2}}\left[ {2mr\sigma}^{2} \right]-\frac{1}{4\left( rm \right)^{2}}\left[ r\left\{ \frac{m-1}{2}\left( {\varphi^{2}}_{1\left( 1 \right)}^{\left( 1 \right)}{+\varphi^{2}}_{2m\left( 2m \right)}^{\left( 2m \right)} \right)+\frac{m+1}{2}\left( {\varphi^{2}}_{m\left( m \right)}^{\left( m \right)}+{\varphi^{2}}_{\left( m+1 \right)\left( m+1 \right)}^{\left( m+1 \right)} \right) \right\} \right]$$

$$=\frac{\sigma^{2}}{2rm}-\frac{1}{4\left( rm \right)^{2}}\left[ \frac{m-1}{2}\left( {\varphi^{2}}_{1\left( 1 \right)}^{\left( 1 \right)}{+\varphi^{2}}_{2m\left( 2m \right)}^{\left( 2m \right)} \right)+\frac{m+1}{2}\left( {\varphi^{2}}_{m\left( m \right)}^{\left( m \right)}+{\varphi^{2}}_{\left( m+1 \right)\left( m+1 \right)}^{\left( m+1 \right)} \right) \right]$$
