# Supplementary material for: Double extreme-cum-median ranked set sampling
Source: PLoS One. 2024 Dec 19;19(12):e0312140. doi: 10.1371/journal.pone.0312140 (PMC11658490; doi:10.1371/journal.pone.0312140)
Supplement: Attachment — Submitted filename: Report1.pdf [file pone.0312140.s003.pdf]

Report of the paper (PONE-D-24-07535R1) entitled 'Double Extreme-Cum-Median Ranked Set Sampling'

by

Muhammad Zubair, et al.

The authors introduced a new improved ranked set sampling procedure named as Double Extreme-cum-Median Ranked Set Sampling (DEMRSS). By simulation, they compared their proposed scheme with some other sampling procedures. Finally, a real data set was analyzed. In my opinion, this paper can not be considered as an interesting paper for some reasons below. So, the publication of this paper is not recommended for the journal of *PLOS ONE*.

(1) The authors should give the number of each section. For example, "1. Introduction" instead of "Introduction" for the title of Section 1.

(2) The authors should give the number of each page.

(3) Page 3, line 8, "see Zamanzade and Mahdizadeh (2020)" should be corrected as "see [21]".

(4) The authors should explain the means of "$y_{i(i)j}, \mu_{y(i)}$" in Equations 1 and 2.

(5) As pointed out by two reviewers, please don't cite so many references together without discussing them as you have given after Equation 2. Some of them can be deleted.

(6) The symbols under Equations 5 and 6 are not clear.

(7) Why the mean estimators under DEMRSS for even and odd sample sizes are unbiased?

(8) How can we obtain Equations 12 and 13?
