# Supplementary material for: Double extreme-cum-median ranked set sampling
Source: PLoS One. 2024 Dec 19;19(12):e0312140. doi: 10.1371/journal.pone.0312140 (PMC11658490; doi:10.1371/journal.pone.0312140)
Supplement: Attachment — Submitted filename: Response to Reviewers.docx [file pone.0312140.s002.docx]

**Replies to Referee’s Comments**

**Double Extreme-Cum-Median Ranked Set Sampling**

We are thankful to the Editor and the anonymous Reviewers for the useful comments and the opportunity to improve our paper. We have revised the article by addressing all the suggested points. Here are the point-by-point responses to all the comments.

**Reviewer #1:**

1) The given literature review is not adequate. Therefore, it is suggested that more studies be added. Specifically, integrating ranked set samplings with SPC techniques is a hot topic. Consequently, I may suggest adding a specific paragraph on the importance or usage of ranked set samplings in control chart studies.

Reply: Thanks for your suggestion. The importance or usage of ranked set samplings in control chart studies is added.

2) The authors provided the references 6 to 25 without giving details of them. Please write the significance of each citation in at least one line.

Reply: Thanks for your suggestion. The details of references are added.

3) The citation style needs to be improved. For example, wherever a line starts with a reference, then reference can be made in the form of Author [number]; a similar form may also follow when a citation is at the end of the line but with the word “by”. For example, ranked set sampling is proposed by Zubair et al. [1].

Reply: Thanks for your suggestion. The citation style is improved.

4) Sections are not adequately divided. I may suggest making sections as (1) Introduction; (2) Mathematical Framework (including ranked set sampling (RSS), Double RSS, Extreme-cum-Median RSS, Proposed sampling Scheme); (3) Design of Simulation Study (including Design parameters for simulations, algorithm of the simulations, Performance Measures); (4) Results and Discussion; (5) Real-life examples and (6) Conclusion and Future Directions.

Reply: Thanks for your suggestion. Sections are properly divided.

5) Provide the algorithm of the simulation study.

Reply: Thanks for your suggestion. Simulation Procedure is provided in detail.

6) Provide a graphical display of how RSS, DRSS, EMRSS, and DEMRSS sampling schemes differ from each other.

Reply: Thanks for your suggestion. Provided in Fig 1 & 2.

7) It is suggested that the manuscript be read and the grammatical errors resolved. Moreover, use the consistent terms. For example, “Weibull distribution” or “Weibull Distribution”

Reply: Thank you for the suggestion. We have carefully checked the language and typo errors in the article.

**Reviewer #2:**

1- Abstract needs a revision, please don’t give abbreviations in abstract and avoid to write in brackets.

Reply: Thanks for your suggestions. We have revised the abstract carefully.

2- Please don’t cite so many references together without discussing them as you have given after equation (2).

Reply: Thanks for your suggestions. The said changes are made.

3- Give a separate table of list of abbreviations and notations. This can be placed in appendix.

Reply: Thanks for your suggestions. Abbreviations and notations are already explained in the manuscript.

4- Literature needs to be arranged in the different sections. According to the heading. Please consider the following papers and cite them in the different sections accordingly.

Noor-ul-Amin, M., Arif, F. & Hanif, M. Modified Extreme Ranked Sets Sampling with Auxiliary Variable. Proc. Natl. Acad. Sci., India, Sect. A Phys. Sci. 91, 537–542 (2021). <https://doi.org/10.1007/s40010-020-00698-6>
Noor-ul-Amin, M., Tayyab, M. Influence on Estimation of Mean by Using Quartile Paired Double Ranked Set Sampling. Proc. Natl. Acad. Sci., India, Sect. A Phys. Sci. 91, 117–121 (2021). <https://doi.org/10.1007/s40010-020-00680-2>
Tayyab, M., Noor-ul-Amin, M. & Hanif, M. Quartile Pair Ranked Set Sampling: Development and Estimation. Proc. Natl. Acad. Sci., India, Sect. A Phys. Sci. 91, 111–116 (2021). <https://doi.org/10.1007/s40010-019-00651-2>

Reply: Thanks for your suggestion. We have cited the said references in the revised manuscript.
