# Supplementary material for: Double extreme-cum-median ranked set sampling
Source: PLoS One. 2024 Dec 19;19(12):e0312140. doi: 10.1371/journal.pone.0312140 (PMC11658490; doi:10.1371/journal.pone.0312140)
Supplement: Attachment — Submitted filename: Response to Reviewers 2.docx [file pone.0312140.s004.docx]

**Replies to Referee’s Comments**

**Double Extreme-Cum-Median Ranked Set Sampling**

We are thankful to the Editor and the anonymous Reviewers for the useful comments and the opportunity to improve our paper. We have revised the article by addressing all the suggested points. Here are the point-by-point responses to all the comments. In the revised version of the paper, we highlighted the changes in yellow color.

**Reviewer #3:**

1) The authors should give the number of each section. For example, “1. Introduction” instead of “Introduction” for the title of Section.

Reply: Thanks for your suggestion. The given suggestion is already incorporated.

2) The authors should give the number of each page

Reply: Thanks for your suggestion. Each page number has been assigned.

3) Page 3, line 8, “see Zamanzade and Mahdizadeh (2020)” should be corrected as “see [21]”.

Reply: Thanks for your suggestion. The said reference is corrected.

4) The authors should explain the means of “yi(i)j, µy(i)” in Equations 1 and 2.

Reply: Thanks for your suggestion. The given terms are properly defined after Equation 1 and 2.

5) As pointed out by two reviewers, please don’t cite so many references together without discussing them as you have given after Equation 2. Some of them can be deleted.

Reply: Thanks for your suggestion. The discussion of the references has been incorporated.

6) The symbols under Equations 5 and 6 are not clear.

Reply: Thanks for your suggestion. The symbols under Equations 5 and 6 are cleared.

7) Why the mean estimators under DEMRSS for even and odd sample sizes are unbiased?

Reply: Thank you for the suggestion. The reference is mentioned after Equation 11.

8) How can we obtain Equations 12 and 13?

Reply: Thank you for the suggestion. The derivations/proves of Equation 12 and 13 are incorporated in Appendix I.
